# 3-(Methylthio)Propionic Acid from *Bacillus thuringiensis* Berliner Exhibits High Nematicidal Activity against the Root Knot Nematode *Meloidogyne incognita* (Kofoid and White) Chitwood

**DOI:** 10.3390/ijms25031708

**Published:** 2024-01-30

**Authors:** Ling Chen, Yueying Wang, Lei Zhu, Yong Min, Yuxi Tian, Yan Gong, Xiaoyan Liu

**Affiliations:** National Biopesticide Engineering Technology Research Centre, Hubei Biopesticide Engineering Research Centre, Hubei Academy of Agricultural Sciences, Wuhan 430064, China; chenling@nberc.com (L.C.); yueying.wang@nberc.com (Y.W.); zhulei@nberc.com (L.Z.); yong.min@nberc.com (Y.M.); tianyuxi@nberc.com (Y.T.); gongyan@nberc.com (Y.G.)

**Keywords:** *Bacillus thuringiensis*, volatile organic compounds, 3-(methylthio)propionic acid, *Meloidogyne incognita*, nematicidal activity, attraction

## Abstract

Root knot nematodes cause serious damage to global agricultural production annually. Given that traditional chemical fumigant nematicides are harmful to non-target organisms and the environment, the development of biocontrol strategies has attracted significant attention in recent years. In this study, it was found that the *Bacillus thuringiensis* Berliner strain NBIN-863 exhibits strong fumigant nematicidal activity and has a high attraction effect on *Meloidogyne incognita* (Kofoid and White) Chitwood. Four volatile organic compounds (VOCs) produced by NBIN-863 were identified using solid-phase microextraction and gas chromatography–mass spectrometry. The nematicidal activity of four VOCs, namely, *N*-methylformamide, propenamide, 3-(methylthio)propionic acid, and phenylmalonic acid, was detected. Among these compounds, 3-(methylthio)propionic acid exhibited the highest direct contact nematicidal activity against *M. incognita*, with an LC50 value of 6.27 μg/mL at 24 h. In the fumigant bioassay, the mortality rate of *M. incognita* treated with 1 mg/mL of 3-(methylthio)propionic acid for 24 h increased to 69.93%. Furthermore, 3-(methylthio)propionic acid also exhibited an inhibitory effect on the egg-hatching of *M. incognita*. Using chemotaxis assays, it was determined that 3-(methylthio)propionic acid was highly attractive to *M. incognita*. In pot experiments, the application of 3-(methylthio)propionic acid resulted in a reduction in gall numbers, decreasing the number of galls per gram of tomato root from 97.58 to 6.97. Additionally, the root length and plant height of the treated plants showed significant increases in comparison with the control group. The current study suggests that 3-(methylthio)propionic acid is a novel nematicidal virulence factor of *B. thuringiensis*. Our research provides evidence for the potential use of NBIN-863 or its VOCs in biocontrol against root knot nematodes.

## 1. Introduction

Plant-parasitic nematodes (PPNs) are distributed globally and can cause USD 80–173 billion in economic losses to agricultural production annually [1,2]. Among the PPNs, root knot nematodes (RKNs, *Meloidogyne* spp.) pose the greatest threat to global food security, as RKNs can infect more than 5500 plant species worldwide, including almost all vascular plants [3,4]. *Meloidogyne incognita* (Kofoid and White) Chitwood is one of the most harmful RKNs. The features of obligate mitotic parthenogenesis and polyploidy enable *M. incognita* to harbor wider geographical distributions and host ranges [5]. RKNs are a kind of sedentary parasitic nematode, only the second-stage juvenile (J2) has the ability to migrate and infect the host [6]. By propelling the stylet to cause physical damage and releasing a series of enzymes and effectors, the infective J2 can migrate to the vascular cylinder and establish permanent feeding sites, ultimately forming numerous galls in the host’s roots [7,8]. The parasitism of RKNs can lead to plant wilting, stunting, leaf chlorosis, and reduced yield [9,10]. Due to their covert parasitic characteristics, controlling RKNs can be challenging [11]. Currently, the main control strategy against RKNs relies on chemical nematicides [12]. However, the use of certain effective chemical nematicides has been strictly banned in some countries due to their high toxicity to non-target organisms and the environment. These banned chemicals include dibromochloropropane, bromomethane, and 1,3-dichloropropene [13,14,15]. In China, although fosthiazate and avermectin are currently the most widely used chemical nematicides, the development of resistance to fosthiazate and the poor mobility of avermectin have led to failures in controlling RKNs in some cases [16]. Therefore, there is an urgent need to develop novel nematicides that are highly effective and environmentally friendly for controlling RKNs.

Previous studies have shown that biological control has great potential to enhance and serve as an alternative to current chemical control strategies [17,18,19,20]. It was reported that certain microorganisms can control and antagonize PPNs through various active modes. For example, *Arthrobotrys oligospora* Fresenius captures nematodes by forming special traps [21]; *Purpureocillium lilacinum* (Thorn) Samson and *Pasteuria penetrans* (Thorne) Sayre and Starr parasitize RKN eggs and J2 by penetrating and infecting them [22,23]; *Bacillus cereus Bacillus cereus* Frankland and Frankland and *Alcaligenes faecalis* Castellani and Chalmers can secrete proteases to kill nematodes directly [24,25]; and *Pseudomonas rhodesiae* Coroler inhibits the parasitism of RKNs through both antagonistic effects and induced plant resistance [26]. Therefore, these nematicidal microorganisms and their active products could serve as valuable resources for the development of new nematicides against RKNs [27].

Among the active metabolites of nematicidal microorganisms, volatile organic compounds (VOCs) have garnered increasing interest due to their high activity [28,29]. In the rhizosphere soil environment where RKNs exist, these VOCs can provide beneficial functions in three ways: they directly inhibit PPNs, they promote plant growth, and they trigger systemic resistance in plants [29,30,31]. Microorganisms often simultaneously produce several nematicidal VOCs against RKNs. For instance, *Pseudomonas putida* (Trevisan) Migula 1A00316 was able to produce five VOCs that exhibited strong direct contact nematicidal activity against *M. incognita* J2 [32]; seven of the ten primary VOCs produced by *Brevundimonas diminuta* (Leifson and Hugh) Segers YMF3.00050 were found to be highly toxic to *Meloidogyne javanica* (Treub) Chitwood [33]; and *Bacillus altitudinis* Shivaji AMCC 1040, isolated from suppressive soils, produced six VOCs that possess nematicidal activity [34]. The currently reported nematicidal VOCs mainly include alcohols, aldehydes, ketones, alkyls, alkenes, esters, ethers, acids, alkynes, heterocyclic, and phenolic compounds [34,35,36,37]. Most of these VOCs have good dispersibility, allowing them to easily penetrate the soil [38]. Furthermore, the use of microbial VOCs in agricultural production could be economically feasible, and their toxicity to humans is lower than that of conventional nematicides [39]. Hence, investigating microbial VOCs with high activity against RKNs will be beneficial for the creation of safer and more efficient nematicides.

*Bacillus thuringiensis* Berliner (*Bt*) is a Gram-positive sporulating bacterium. During the sporulation phase, it can produce insecticidal and nematicidal parasporal crystals [40]. As a nematode pathogen, *Bt* can produce a range of nematicidal virulence factors, such as crystal proteins, metalloproteinase, collagenase, and so on [41,42,43,44]. However, only a few studies have described the nematicidal VOCs produced by *Bt*. It is not yet clear whether this typical nematode pathogen can produce nematicidal VOCs. In this study, we found that the *B. thuringiensis* strain NBIN-863 exhibited high nematicidal fumigant activity against *M. incognita*, and *M. incognita* J2 was highly attracted to the strain’s fermentation broth. We further identified the VOCs produced by *B. thuringiensis* NBIN-863. By testing the nematicidal activities of the purified volatiles, we found that 3-(methylthio)propionic acid was the most potent compound among the identified VOCs. Finally, the antagonistic effect of 3-(methylthio)propionic acid against *M. incognita* in pots was detected. The results of this study suggest that 3-(methylthio)propionic acid is a novel virulence factor of *Bt*, highlighting the potential of *B. thuringiensis* NBIN-863 as a promising candidate for bionematicides.

## 2. Results

### 2.1. Nematicidal Effects of B. thuringiensis NBIN-863 against M. incognita

**Figure 1:**
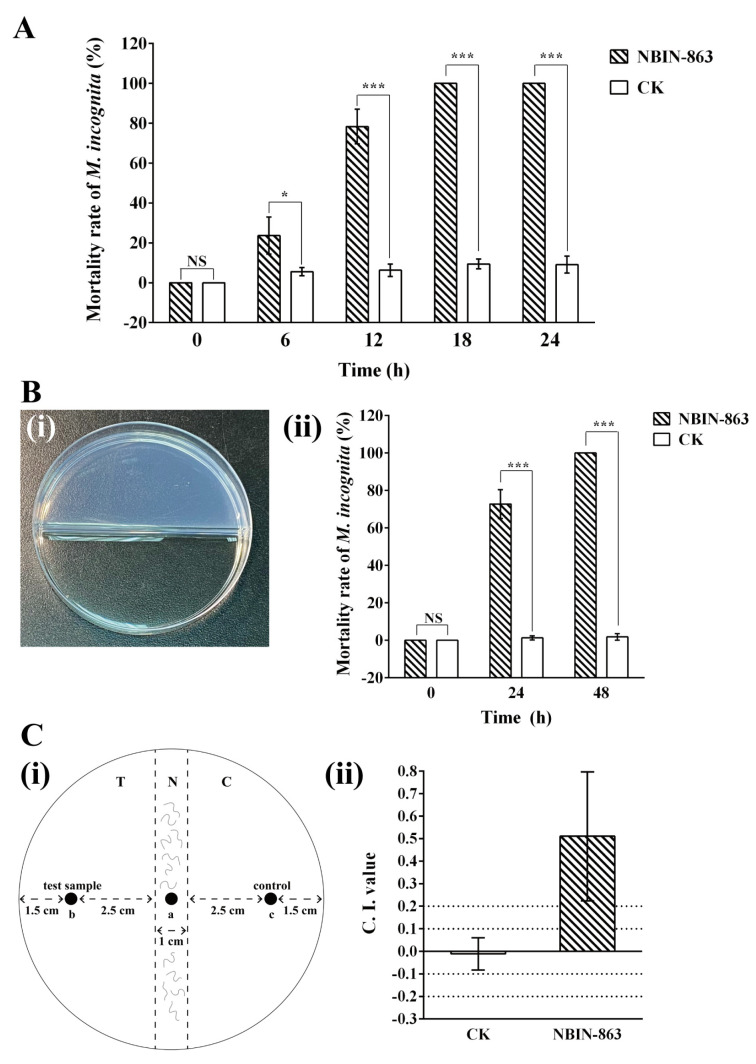
Nematicidal effects of *B. thuringiensis* NBIN-863 against *M. incognita*. (**A**) Direct contact nematicidal activity of the fermentation supernatant of NBIN-863 against *M. incognita* J2. (**B**) Fumigant activity of NBIN-863 against *M. incognita* J2: (**i**) schematic showing the two-compartment plate assay and (**ii**) the mortality rate of nematodes in the chamber adjacent to the chamber receiving samples. (**C**) Attractant effect of NBIN-863 on *M. incognita* J2: (**i**) schematic of the chemotaxis assay for *M. incognita* in a Petri dish (diameter 9 cm) and (**ii**) chemotactic response of *M. incognita* J2 to the fermentation broth of NBIN-863. Among these experiments, the fermentation medium was used as the negative control (CK). In (**A**,**B**), each column represents the mean ± standard deviation of three biological replicates. In (**C**), the chemotaxis index (C. I.) values are presented as means ± standard deviation for six biological replicates. Asterisks above the bars indicate significant differences according to one-way ANOVA with Tukey’s HSD tests (* *p* < 0.05 and *** *p* < 0.001), and NS indicates no significant difference.

With the 96-well plate bioassay, it was shown that the fermentation supernatant of *B. thuringiensis* NBIN-863 exhibited high direct-contact nematicidal activity against *M. incognita* J2. The result demonstrated that as the treatment time increased, the mortality rate of nematodes gradually increased, and after 18 h of treatment, the mortality rate of nematodes reached 100% (Figure 1A).

The fumigant activity of NBIN-863 was also detected using the two-compartment plate assay. The result showed that after adding the fermentation supernatant of NBIN-863 to one compartment for 24 h, the mortality rate of *M. incognita* in the other compartment reached 72.7%. After further treatment for 48 h, the nematode mortality rate reached 100% (Figure 1B).

Furthermore, to determine the attractant or repellent effect of NBIN-863 on *M. incognita*, a chemotaxis assay was conducted. The result showed that *M. incognita* J2 was highly attracted (C. I. value ≥ 0.2) to the fermentation broth of NBIN-863, while in the control treatment, the nematodes exhibited a random response (−0.1 ≤ C. I. value < 0.1) (Figure 1C). The results suggested that the NBIN-863 culture might attract and kill *M. incognita* by producing volatiles.

### 2.2. Identification of the VOCs Produced by B. thuringiensis NBIN-863

Using solid-phase microextraction (SPME) and gas chromatography–mass spectrometry (GC-MS) analysis, the VOCs in the fermentation supernatant of NBIN-863 were identified. Four differential peaks of VOCs were observed in the total ion current chromatograms, distinguishing the fermentation supernatant from the control medium (Figure S1). By comparing the mass spectrum of the substance with the GC-MS system database (NIST 08), four volatiles, namely, *N*-methylformamide, propenamide, 3-(methylthio)propionic acid, and phenylmalonic acid, were identified in the fermentation supernatant of NBIN-863 (Table 1). To carry out further experiments, we purchased the four commercially purified compounds.

### 2.3. Nematicidal Activity of 3-(Methylthio)Propionic Acid against M. incognita

**Figure 2:**
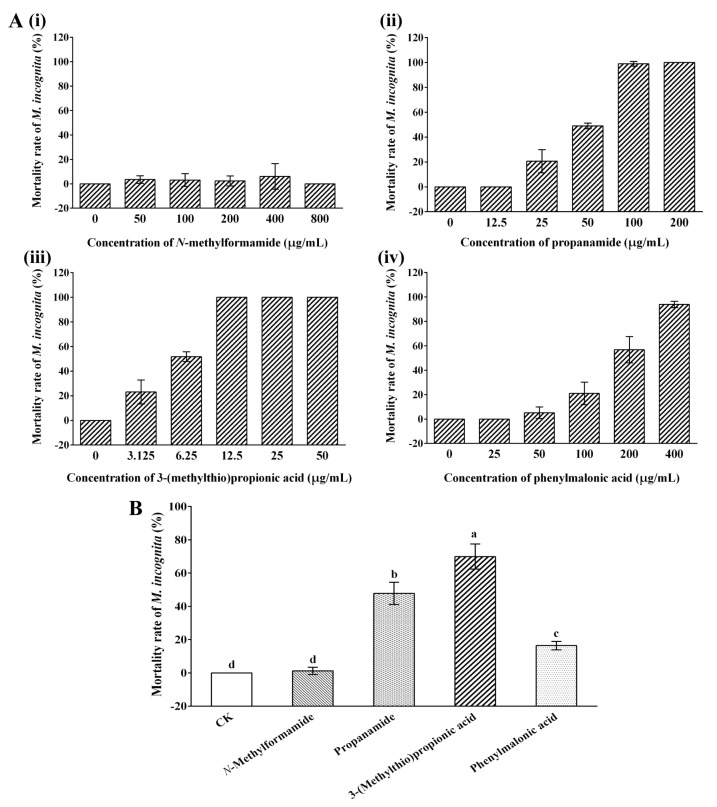
Nematicidal activity of the VOCs against *M. incognita*. (**A**) The direct contact nematicidal activity of *N*-methylformamide (**i**), propenamide (**ii**), 3-(methylthio)propionic acid (**iii**), and phenylmalonic acid (**iv**) against *M. incognita* J2 was detected after 24 h. (**B**) The fumigant activity of the VOCs against *M. incognita* J2 was detected after 24 h. Distilled water was used as the negative control (CK). Each column represents the mean ± standard deviation of three biological replicates. Letters indicate significant differences among the different treatments according to one-way ANOVA with Tukey’s HSD tests (*p* < 0.05).

Given that the strain NBIN-863 produced four VOCs that may have nematicidal activity, a nematicidal bioassay was performed using commercially available compounds. The direct contact nematicidal activity of the four VOCs is illustrated in Figure 2A. After 24 h of treatment, the four VOCs showed varying levels of nematicidal activity against *M. incognita* J2. As the concentration increased, the mortality rate of nematodes treated with propenamide, 3-(methylthio)propionic acid, and phenylmalonic acid increased gradually. However, even when the concentration of *N*-methylformamide reached 800 μg/mL, there were still almost no dead nematodes in the treatment group. Based on the experimental data, it was demonstrated that 3-(methylthio)propionic acid exhibited the highest direct-contact nematicidal activity, with an LC_50_ value of 6.27 μg/mL at 24 h. This was followed by propenamide and phenylmalonic acid, with LC_50_ values of 53.99 μg/mL and 211.92 μg/mL at 24 h, respectively. The LC_50_ value of *N*-methylformamide was not evaluated due to its weak nematicidal activity. The fumigant activity of volatiles against *M. incognita* was also assessed. It was shown that when the concentration of volatiles was 1 mg/mL, the mortality rates of nematodes treated with *N*-methylformamide, propenamide, 3-(methylthio)propionic acid, and phenylmalonic acid for 24 h were 1.23%, 47.83%, 69.93%, and 16.44%, respectively (Figure 2B). The results indicate that 3-(methylthio)propionic acid and propenamide exhibited relatively strong fumigant nematicidal activity, while phenylmalonic acid showed relatively weak fumigant activity. *N*-methylformamide demonstrated no fumigant activity against *M. incognita* under the present conditions.

**Figure 3:**
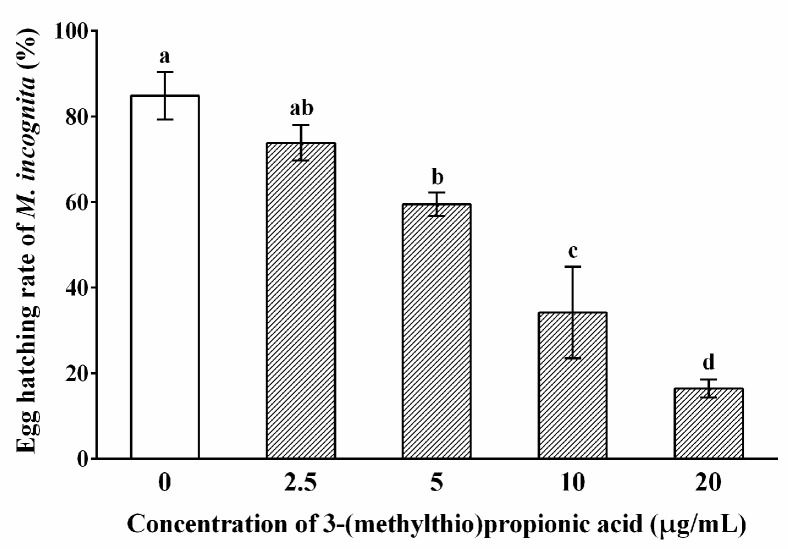
Inhibitory effect of 3-(methylthio)propionic acid on the egg-hatching of *M. incognita*. The egg-hatching rate values are presented as means ± standard deviation for six biological replicates. Letters indicate significant differences among the different treatments according to one-way ANOVA with Tukey’s HSD tests (*p* < 0.05).

The results demonstrated that 3-(methylthio)propionic acid was the most active compound among the four VOCs. To further investigate the nematicidal effect of 3-(methylthio)propionic acid, we detected its inhibitory effect on the egg-hatching of *M. incognita* (Figure 3). It was demonstrated that after 10 days, as the concentration of 3-(methylthio)propionic acid increased, there was a gradual and significant decrease in the egg-hatching rate compared with the control group (treated with distilled water). When the concentration of 3-(methylthio)propionic acid was 5 μg/mL, the egg hatch rate decreased significantly to 59.49% (*p* < 0.05), whereas the egg hatch rate of the control treatment was 84.90%. Moreover, only 16.45% of the eggs hatched when treated with 20 μg/mL of 3-(methylthio)propionic acid. The result indicates that 3-(methylthio)propionic acid was also toxic to the eggs of *M. incognita*.

### 2.4. Chemotaxis of M. incognita toward 3-(Methylthio)Propionic Acid

**Figure 4:**
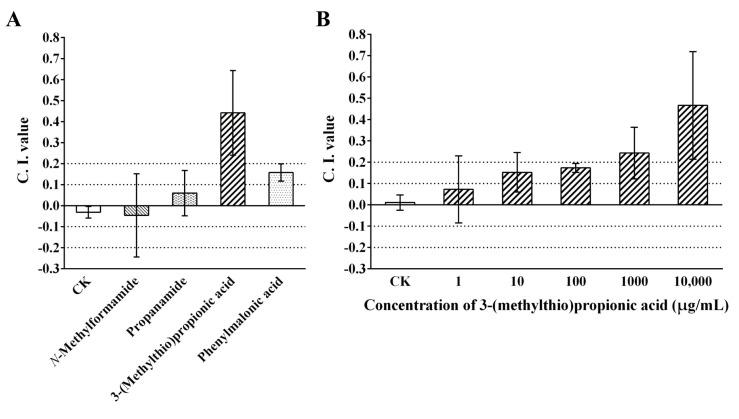
The attractant effect of VOCs on *M. incognita*. (**A**) Chemotactic response of *M. incognita* J2 to the four VOCs produced by *B. thuringiensis* NBIN-863. (**B**) Chemotactic response of *M. incognita* J2 to 3-(methylthio)propionic acid with a concentration gradient. Distilled water was used as the negative control (CK). The chemotaxis index (C. I.) values are presented as means ± standard deviation for six biological replicates.

The results of the chemotaxis assay with the four VOCs at high concentrations showed that 3-(methylthio)propionic acid was highly attractive to *M. incognita* J2 (C. I. value ≥ 0.2), phenylmalonic acid was slightly attractive to J2 (0.1 ≤ C. I. value < 0.2), while worms treated with *N*-methylformamide or propenamide exhibited a random response (−0.1 ≤ C. I. value < 0.1) (Figure 4A). In addition, the chemotactic response of *M. incognita* J2 to 3-(methylthio)propionic acid with gradient concentrations was also investigated due to its high attractiveness (Figure 4B). It was shown that 3-(methylthio)propionic acid could attract nematodes at concentrations of 10 mg/mL, 1 mg/mL, 100 μg/mL, and 10 μg/mL with a C. I. value of 0.467, 0.243, 0.174, and 0.153, respectively. This result implies that the attraction of 3-(methylthio)propionic acid to *M. incognita* is dose-dependent.

### 2.5. Antagonistic Effect of 3-(Methylthio)Propionic Acid against M. incognita in Pot Experiments

**Figure 5:**
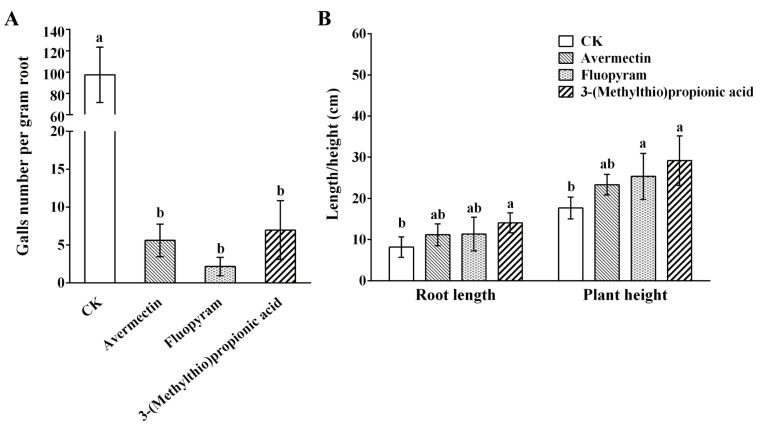
Potted plant control effect of 3-(methylthio)propionic acid against *M. incognita*. (**A**) Nematode infection of tomato plants under various treatments. (**B**) Growth of tomato plants under various treatments. In pot experiments, water was used as the negative control (CK), and avermectin and fluopyram were used as the positive controls. Each column represents the mean ± standard deviation of six biological replicates. Letters indicate significant differences among the different treatments according to one-way ANOVA with Tukey’s HSD tests (*p* < 0.05).

To detect the antagonistic effect of 3-(methylthio)propionic acid against *M. incognita* infection, pot experiments were conducted using tomato plants. The results at 60 dpi (days post-infection) showed that the average galls number per gram root of plants treated with 3-(methylthio)propionic acid decreased to 6.97, whereas the galls number of plants in the negative control (treated with distilled water) was 97.58 (Figure 5A). The results also indicated that the control efficacy of 3-(methylthio)propionic acid on potted plants was comparable to that of avermectin and fluopyram, which are widely used commercial nematicides. Furthermore, plant growth under different treatments was observed. The results demonstrated that under greenhouse conditions, the average root length and plant height of plants treated with 3-(methylthio)propionic acid were 14.05 cm and 23.33 cm, respectively. These measurements showed a significant increase of 71.97% and 32.03% compared with the control (Figure 5B). It is implied that 3-(methylthio)propionic acid might promote the growth of tomato plants.

## 3. Discussion

The *Bt* pesticide is one of the most successful bacterial agents, with a safety record in agriculture that spans more than half a century [45,46]. The fermentation technology and production process of *Bt* are relatively well-established. These factors are advantageous for developing *Bt* as nematicidal products. However, although *Bt* was reported to have nematicidal activity against RKNs as early as 1972, there are currently very few *Bt* nematicides available [47]. Most of the virulence factors of *Bt* were reported to function on nematode intestines, so their nematicidal activity depends on nematode feeding [48,49,50]. However, RKNs, as obligate endoparasitic nematodes, only feed through feeding tubes within the host’s roots [51]. This situation may lead to the unstable effectiveness of certain nematicidal *Bt* strains and limit the development of *Bt* nematicidal products. The current study indicated that *B. thuringiensis* NBIN-863 exhibits high nematicidal activity against *M. incognita* by producing nematicidal VOCs. In contrast to most of the known virulence factors of *Bt*, these VOCs could rapidly kill nematodes through direct contact or fumigation. This study is the first to report that *Bt* can produce nematicidal VOCs and has discovered a novel type of nematicidal virulence factor in *Bt*. Our research not only enhances the understanding of *Bt* as a nematode pathogen but also highlights the significant potential of *B. thuringiensis* NBIN-863 as a promising candidate for bionematicides because of its rapid-killing activity against RKNs.

In the bioassay experiments with *B. thuringiensis* NBIN-863, the fermentation broth exhibits high nematicidal activity. Additionally, it attracted *M. incognita* J2 to move toward the strain’s location. Subsequent bioassays with the VOCs suggested that NBIN-863 emits VOCs, including 3-(methylthio)propionic acid and phenylmalonic acid, to attract nematodes and then kill them by producing nematicidal VOCs, such as 3-(methylthio)propionic acid, propenamide, and phenylmalonic acid. It is speculated that *B. thuringiensis* NBIN-863 could antagonize RKNs through an attracting-and-killing mode. This mode is somewhat similar to the Trojan horse mechanism of *Bacillus nematocida* Huang B16 [52]. The strain B16 can produce seven VOCs to lure nematodes and secrete two proteases to kill nematodes. The nematophagous fungus *Pochonia chlamydosporia* (Goddard) Zare and Gams has also been found to exhibit a similar mode of attraction and toxicity [53]. In the present study, the VOCs produced by NBIN-863 serve a dual function of attracting and being toxic to *M. incognita*. The intriguing attracting-and-killing mode of NBIN-863 will be thoroughly investigated in our future work.

The VOCs produced by bacteria of the genus *Bacillus* play an important role in the field of biological control [54]. *Bacillus* species can produce various VOCs that target different organisms, including bacteria, fungi, and nematodes [55,56,57]. However, in *Bt* species, only antifungal VOCs have been reported to date [54,58,59,60]. Interestingly, the reported VOCs produced by *Bt* were not detected in our study. It is inferred that some VOCs may not be identified during the extraction process due to their low abundance or other technical factors, such as the influence of cultivation conditions and limitations of extraction methods. Among the four VOCs produced by *B. thuringiensis* NBIN-863, 3-(methylthio)propionic acid, propenamide, and phenylmalonic acid exhibit varying levels of nematicidal activity against RKNs, 3-(methylthio)propionic acid and phenylmalonic acid were found to be attractive to *M. incognita* J2, while *N*-methylformamide appears to have no activity against nematodes. Given that the mixture of VOCs produced by bacteria may have a stronger control effect against nematodes than treatment with a single compound, the multi-component combination of the four VOCs should be tested for their activity against nematodes in future research [61]. Perhaps the function of *N*-methylformamide could be identified during the nematode bioassays involving the multi-component combination.

Organic acids produced by bacteria are an important natural resource for PPN control [36,62]. Our data demonstrated that 3-(methylthio)propionic acid exhibited the strongest direct contact and fumigant nematicidal activity against *M. incognita* and was highly attractive to *M. incognita* J2. To assess the nematicidal activity of 3-(methylthio)propionic acid more effectively, we compared its nematicidal activity with that of other structural analogs. Four compounds, namely, 3-methoxypropionic acid, valeric acid, isovaleric acid, and L-cysteine, were used to detect their direct-contact nematicidal activity (Figure S2). The results showed that 3-methoxypropionic acid, valeric acid, and isovaleric acid were also toxic to *M. incognita* J2, with LC_50_ values of 36.49 μg/mL, 23.21 μg/mL, and 56.33 μg/mL at 24 h, respectively (data shown in Figure S2). The data illustrated that 3-(methylthio)propionic acid was the most active compound among the five compounds. At low concentrations, 3-(methylthio)propionic acid was significantly more toxic to nematodes than the other compounds. The methylthio group is speculated to contribute to the nematicidal activity of 3-(methylthio)propionic acid, making its toxicity against *M. incognita* stronger than that of other structural analogs. Furthermore, after the examination of morphological changes in *M. incognita* J2 following treatment with 3-(methylthio)propionic acid, we observed complete damage to the intestinal tissue of the J2 (Figure S3). Similar phenotypes of J2 were reported in a previous study, which identified the nematicidal activity of acetaldehyde and dimethyl disulfide produced by *Virgibacillus dokdonensis* Yoon [11]. However, research on the nematicidal mechanism of VOCs is relatively limited, and the molecular targets of most VOCs are unclear [36,63]. To effectively utilize 3-(methylthio)propionic acid for controlling RKNs, additional research is required to uncover its mechanism of action.

In pot experiments, it was found that 3-(methylthio)propionic acid could significantly decrease the number of galls on tomato roots. The control efficacy of 3-(methylthio)propionic acid against *M. incognita* was similar to that of the primary commercial chemical nematicides currently in use. Additionally, we discovered that the root length and plant height of tomatoes treated with 3-(methylthio)propionic acid were significantly higher than that of tomatoes in the control group. The promoting effect of 3-(methylthio)propionic acid on plant growth needs to be verified with further experiments. Nevertheless, the results of our pot experiments demonstrated the significant potential of 3-(methylthio)propionic acid as a novel nematicide. The efficacy of 3-(methylthio)propionic acid in controlling RKNs should be further evaluated in the field.

## 4. Materials and Methods

### 4.1. Bacterial Strains and Culture Conditions

The *B. thuringiensis* strain NBIN-863 was isolated from soil collected from Jiuhua Mountain in the Anhui Province of China and stored in our laboratory and the China Center for Type Culture Collection (CCTCC Number: M2013612). A single clone of strain NBIN-863 was inoculated into Luria–Bertani (LB) medium (1% tryptone, 0.5% yeast extract, and 1% NaCl) and incubated at 30 °C with shaking at 220 rpm overnight. When the bacterial concentration reached 10^9^ CFU/mL, 1 mL of seed fluid was inoculated into 100 mL of fermentation medium (3% soybean meal, 1.5% corn steep liquor, and 1.5% corn starch) and incubated in a rotary shaker (220 rpm) at 30 °C for 24 h. The fermentation supernatant of NBIN-863 was obtained by centrifuging the fermentation broth at 12,000 rpm for 2 min. The fermentation broth and supernatant were used for subsequent experiments.

### 4.2. Nematode Material

A population of *M. incognita* was maintained on susceptible tomato plants in the greenhouse at the National Biopesticide Engineering Technology Research Center in Wuhan, China. After 2 months of cultivation at 25 °C, the plants infested with nematodes were uprooted, and roots containing numerous galls and egg masses were cut into small pieces with a diameter of 0.5 cm. The pieces were transferred to a 1% sodium hypochlorite solution and stirred at 1000 rpm for 8 min. The suspension was then sieved through 18-, 60-, 100-, 200-, and 500-mesh sieves in succession and rinsed five times with distilled water. The eggs were collected from the 500-mesh sieve and used for subsequent experiments. The second-stage juveniles (J2) were obtained by incubating the eggs on a 500-mesh sieve at 25 °C, following the method described by Dai et al. [5].

### 4.3. Identification of VOCs of the Bacterial Fermentation Supernatant

To identify the VOCs produced by the strain NBIN-863, solid-phase microextraction (SPME) and gas chromatography–mass spectrometry (GC-MS) were used as described by Huang et al. [11]. The VOCs were identified by comparing the mass spectra of the substances with the mass spectra of the standards in the GC-MS system in the National Institute of Standards and Technology (NIST 08) database. The VOCs in the non-inoculated fermentation medium were used as the control. Each sample was carried out with six replicates.

### 4.4. Commercial Volatiles Compounds

Four commercial volatile compounds were used to detect the nematicidal activity of VOCs: *N*-methylformamide (99%; Aladdin, Shanghai, China), propenamide (97%; Macklin, Nanjing, China), 3-(methylthio)propionic acid (98%; Macklin, Shanghai, China), and phenylmalonic acid (98%; Aladdin, China). Compounds with a similar structure to 3-(methylthio)propionic acid were used to analyze their differences in nematicidal activity: 3-methoxypropionic acid (98%; Macklin, China), L-cysteine (98.5%; Sangon Biotech, Shanghai, China), valeric acid (99%; Macklin, China), and isovaleric acid (99.5%; Aladdin, China). The volatile compounds and structural analogs of 3-(methylthio)propionic acid were diluted with distilled water for subsequent experiments.

### 4.5. Direct Contact Nematicidal Bioassay of the Fermentation Supernatant and VOCs of NBIN-863

The direct contact nematicidal bioassay was performed using 96-well plates. First, 90 μL of a sample containing various concentrations and compounds were added to the wells, respectively, then 10 μL of nematode suspension containing approximately 30 J2 of *M. incognita* was added to each well. After incubating at 25 °C, the state of the nematodes in the well was observed using an inverted microscope (CKX41, Olympus, Hamburg, Germany). The worms were touched with a needle, and if no movement was observed after 2 s, they were considered dead. The supernatant of the fermentation medium was used as the negative control for the bioassay of the fermentation supernatant of NBIN-863. Distilled water was used as the negative control for the bioassay of the VOCs. Each treatment was carried out with three replicates. The mortality rate values of *M. incognita* were adjusted by excluding natural deaths in the negative control, following Schneider–Orelli’s formula [64] as shown below:

Mortality rate = (mortality rate in treatment − mortality rate in control)/(100 − mortality rate in control) × 100%(1)

### 4.6. Fumigant Nematicidal Bioassay of the Fermentation Supernatant and VOCs of NBIN-863

The fumigant nematicidal bioassay was conducted using two-compartment plates (Figure 1B(i)). A total of 1 mL of the fermentation supernatant or VOC solution was added to one compartment. The concentration of VOCs was diluted to 1 mg/mL. Then, 200 μL of nematode suspension containing approximately 100 J2 of *M. incognita* was added to the 2% water–agar medium in the other compartment. All plates were immediately sealed with Parafilm. Then, the plates were incubated at 25 °C in the dark. After incubation for 24 h or 48 h, the mortality rate of *M. incognita* was investigated using an inverted microscope (CKX41, Olympus). The supernatant of the fermentation medium was used as the negative control for the bioassay of the fermentation supernatant of NBIN-863. Distilled water was used as the negative control for the bioassay of the VOCs. Each treatment was carried out with three replicates.

### 4.7. Chemotaxis of M. incognita toward the Fermentation Broth and VOCs of NBIN-863

The chemotaxis assay (Figure 1C(i)) was conducted following previously described methods with some modifications [53,65]. First, 10 mL of 2% water–agar medium was added to Petri dishes with a 9 cm diameter. Then, the plates were divided into three areas: the central part of the plate was defined as the neutral area (N); the side where the test sample was added was defined as the test area (T); and the other side where the control sample was added was defined as the control area (C). A total of 20 μL of nematode suspension containing approximately 100 J2 of *M. incognita* was placed in position a of area N. For the chemotaxis assay of the fermentation broth of NBIN-863, 50 μL of fermentation broth of NBIN-863 was placed in position b of area T, and 50 μL of fermentation medium was placed in position c of area C. For the chemotaxis assay of VOCs, 50 μL of VOC solution was placed in position b of area T, and 50 μL of distilled water was placed in position c of area C. The distance from positions b and c to the edges of area N was 2.5 cm. The plates were sealed with Parafilm and incubated at 25 °C in the dark. After 16 h, the number of worms in area T and area C was counted with an inverted microscope (CKX41, Olympus). The chemotaxis index (C. I.) value was calculated according to the following formula [66]:

C. I. value = (the number of worms in area T − the number of worms in area C)/(the number of worms in area T + the number of worms in area C)(2)

If the C. I. value was ≥ 0.2, the test sample was considered highly attractive; if 0.1 ≤ C. I. value < 0.2, the test sample was considered slightly attractive; if −0.1 ≤ C. I. value < 0.1, the test sample was considered a random response of nematodes; if −0.2 < C. I. value < −0.1, the test sample was considered repellent; if the C. I. value was ≤ −0.2, the test sample was considered highly repellant [67]. The experiment was repeated twice and carried out in triplicate.

### 4.8. Inhibitory Effect of 3-(Methylthio)Propionic Acid on Egg Hatching of M. incognita

Various concentrations of 3-(methylthio)propionic acid solutions were added to the wells of a 96-well plate. Subsequently, 10 μL of egg suspension containing approximately 100 eggs of *M. incognita* was added to each well. The final concentrations of 3-(methylthio)propionic acid were set at 2.5, 5, 10, and 20 μg/mL. Distilled water was used as the negative control. The total number of eggs in each well was counted using an inverted microscope (CKX41, Olympus). The 96-well plates were then incubated at 25 °C in the dark. After an incubation period of 10 days, the number of hatched J2 was quantified. The egg-hatching rate was calculated by dividing the number of hatched J2 by the total number of eggs. The experiment was repeated twice and carried out in triplicate.

### 4.9. Efficacy of 3-(Methylthio)Propionic Acid against M. incognita in the Pot Experiment

To assess the antagonistic effect of 3-(methylthio)propionic acid against *M. incognita* infecting the host, pot experiments with tomato plants were conducted under greenhouse conditions. The seeds of susceptible tomatoes (Jinpeng No. 3, Xi’an Jinpeng Seed Co., Ltd., Xi’an, China) were planted in seedling pots filled with sterilized potting soil. After two weeks, the seedlings were transplanted into 14 cm diameter pots filled with a sterilized sand and soil mixture (*v*/*v*: 1:1). The transplants were kept at 24 °C with a light/dark cycle of 16 h light and 8 h dark and at 60% relative humidity. The two-week-old seedlings were inoculated with 3000 J2 of *M. incognita* per pot. One day after inoculating the nematodes, the potted plants were treated with four different treatments for root irrigation. The four treatments included distilled water, 3-(methylthio)propionic acid, avermectin, and fluopyram. Among them, distilled water was used as the negative control, while avermectin and fluopyram were used as positive controls. The volume of the solutions used for root irrigation was 50 mL. The concentration of 3-(methylthio)propionic acid was diluted to 1 mg/mL. Avermectin (1.8%, Anhui Huaxing Chemical Industry Co., Ltd., Hefei, China) and fluopyram (41.7%, Bayer, Leverkusen, Germany) were diluted for irrigation according to the manufacturer’s protocol. The final concentrations of avermectin and fluopyram used for the pot experiments were calculated to be 0.09 mg/mL and 0.25 mg/mL, respectively. The four treatments for potted plants were prepared, with six replicates for each treatment. The plants were then placed in a greenhouse and watered every two days. At 60 dpi (days post-infection), the plants were carefully uprooted and washed to remove the soil. The gall number was counted, and the root length, root weight, and plant height were measured using a ruler. The infection levels of *M. incognita* were represented by the gall number per gram root.

### 4.10. Data Analysis

All data were reported as the mean ± standard deviation from three or more biological replicates. The differences among treatments were analyzed using one-way ANOVA with Tukey’s HSD test (*p* < 0.05) using SPSS Statistics v22.0 (IBM, Armonk, NY, USA). The data were presented using GraphPad Prism 6.

## 5. Conclusions

In summary, this study revealed the attracting-and-killing activity of *B. thuringiensis* NBIN-863 against *M. incognita* and identified the presence of three nematicidal VOCs produced by NBIN-863. Among these VOCs, 3-(methylthio)propionic acid exhibits the highest nematicidal and attractive activity toward *M. incognita*. Additionally, pot experiments illustrated the significant potential application of 3-(methylthio)propionic acid. Our work not only establishes the role of 3-(methylthio)propionic acid as a novel nematicidal virulence factor of *Bt*, but also provides evidence for the potential utilization of NBIN-863 or its VOCs for biocontrol against RKNs.

## Figures and Tables

**Table 1:** Area percentage of the specific VOCs in the fermentation broth of *B. thuringiensis* NBIN-863 compared to the fermentation medium.

Peak Number	Retention Time (min)	Compound	CAS Number	Area Percentage(%)
6	12.43	*N*-Methylformamide	123-39-7	61.78
7	12.64	Propanamide	79-05-0	4.57
11	15.77	3-(Methylthio)propionic acid	646-01-5	7.32
14	17.75	Phenylmalonic acid	2613-89-0	5.48

## Data Availability

Data are contained within the article and Supplementary Materials.

## Supplementary Materials

The supporting information can be downloaded at: https://www.mdpi.com/article/10.3390/ijms25031708/s1.
