# Supplementary material for: 3-(Methylthio)Propionic Acid from Bacillus thuringiensis Berliner Exhibits High Nematicidal Activity against the Root Knot Nematode Meloidogyne incognita (Kofoid and White) Chitwood
Source: Int J Mol Sci. 2024 Jan 30;25(3):1708. doi: 10.3390/ijms25031708 (PMC10855422; doi:10.3390/ijms25031708)
Supplement: Supplementary file 1 [file ijms-25-01708-s001.zip › ijms-2813593-supplementary.pdf]

Supplementary Materials

3-(Methylthio)propionic acid of *Bacillus thuringiensis* exhibits high nematicidal activity against root knot nematode *Meloidogyne incognita*

Ling Chen, Yueying Wang, Lei Zhu, Yong Min, Yuxi Tian, Yan Gong and Xiaoyan Liu

Materials and Methods in Supplementary Materials

Commercial compounds

Compounds with a similar structure to 3-(methylthio)propionic acid were used to analyze their differences in nematicidal activity: 3-methoxypropionic acid (98%; Macklin, China); L-cysteine (98.5%; Sangon Biotech, China); valeric acid (99%; Macklin, China); and isovaleric acid (99.5%; Aladdin, China). The structural analogues of 3-(methylthio)propionic acid were diluted with distilled water for subsequent experiments.

Direct-contact nematicidal bioassay of the structural analogues of 3-(methylthio)propionic acid

The direct-contact nematicidal bioassay was performed by using 96-well plates. 90 μL of a sample containing various concentrations of compounds were added to the wells, respectively, then 10 μL of nematode suspension containing approximately 30 J2 of *M. incognita* was added to each well. After incubating at 25°C, the state of the nematodes in the well was observed using an inverted microscope (CKX41, Olympus). The worms were touched with a needle, and if no movement was observed after 2 seconds, they were considered dead. Distilled water was used as the negative control. Each treatment was carried out with three replicates. The mortality rate values of *M. incognita* were adjusted by excluding natural deaths in the negative control, following the formula as shown below:

$$\text{Mortality rate} = (\text{mortality rate in treatment} - \text{mortality rate in control}) / (100 - \text{mortality rate in control}) \times 100\% \quad (1)$$

Figures in Supplementary Materials

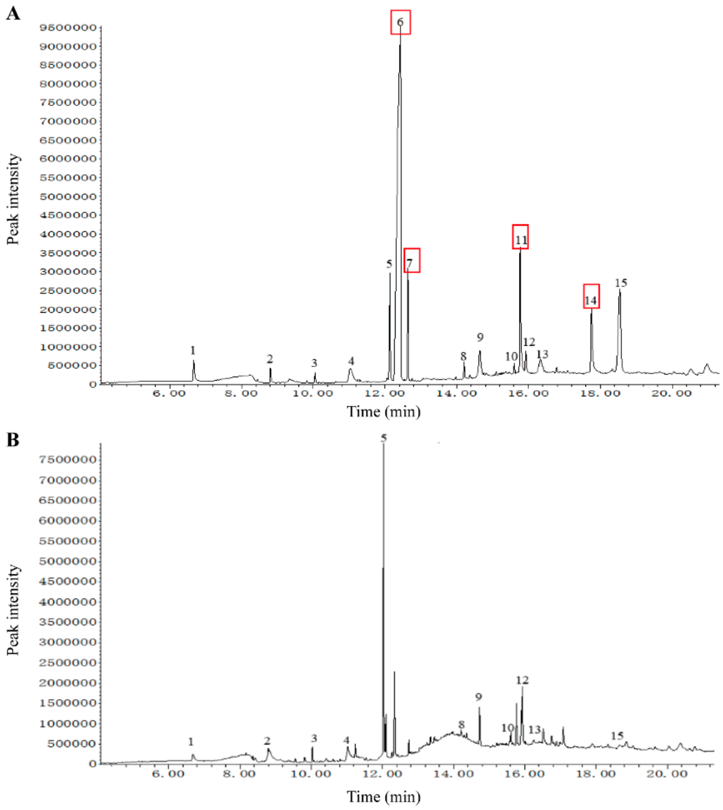

Figure S1. Chromatograms of VOCs from *B. thuringiensis* NBIN-863. The VOCs were isolated using solid-phase microextraction (SPME) and analyzed via gas chromatography-mass

spectrometry (GC-MS). (**A**) Fermentation broth of *B. thuringiensis* NBIN-863. (**B**) Fermentation medium for the control. The specific VOCs peaks in the fermentation broth compared to the fermentation medium were marked in red boxes.

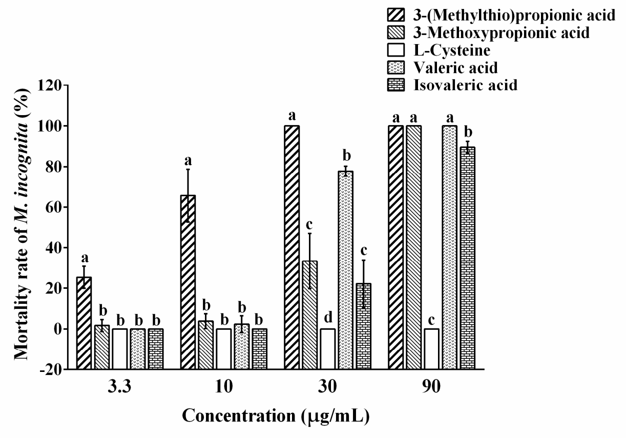

Figure S2. Direct-contact nematicidal activity of structural analogues of 3-(methylthio)propionic acid against *M. incognita* J2 after treatment for 24 h. Each column represents the means ± standard deviation of three biological replicates. Letters indicate significant differences among the different treatments according to one-way ANOVA with Tukey's HSD tests ($P < 0.05$).

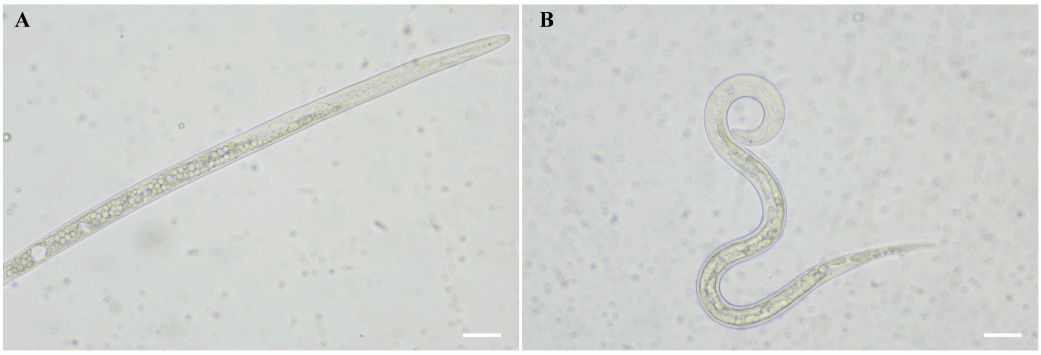

Figure S3. Morphological variations in *M. incognita* J2 after 3-(methylthio)propionic acid treatment. (A) J2 treated with 20 μg/mL 3-(methylthio)propionic acid for 24 h. (B) J2 treated with distilled water for 24 h. Scale bar = 25 μm.
